# Epigenetic Mechanisms Impacting Aging: A Focus on Histone Levels and Telomeres

**DOI:** 10.3390/genes9040201

**Published:** 2018-04-09

**Authors:** Shufei Song, F. Brad Johnson

**Affiliations:** 1 Biochemistry and Molecular Biophysics Graduate Group, Biomedical Graduate Studies, University of Pennsylvania, Philadelphia, PA 19104, USA; sophie.song0822@gmail.com; 2 Department of Pathology and Laboratory Medicine, and Institute on Aging, Perelman School of Medicine, University of Pennsylvania, Philadelphia, PA 19104, USA

**Keywords:** aging, chromatin, epigenetics, histones, telomeres

## Abstract

Aging and age-related diseases pose some of the most significant and difficult challenges to modern society as well as to the scientific and medical communities. Biological aging is a complex, and, under normal circumstances, seemingly irreversible collection of processes that involves numerous underlying mechanisms. Among these, chromatin-based processes have emerged as major regulators of cellular and organismal aging. These include DNA methylation, histone modifications, nucleosome positioning, and telomere regulation, including how these are influenced by environmental factors such as diet. Here we focus on two interconnected categories of chromatin-based mechanisms impacting aging: those involving changes in the levels of histones or in the functions of telomeres.

## 1. An Overview of Aging Mechanisms

Aging is one of the major causes of death in the developed world, even though it is rarely recognized as such. It is usually some other cause that is written down on death certificates, e.g., heart failure, stroke, cancer, pneumonia, or respiratory failure. However, there is no doubt that these pathologies are brought on largely by underlying age-related biological changes.

The question of why we age is still the subject of vigorous debate. Aging occurs in most species, although the fact that some show few or no signs of aging indicates that it is not necessarily inevitable [1]. Historically, aging theories have often been divided into two seemingly opposing categories, invoking either biological programs or random damage. Programmed aging theories include those postulating that individual organisms are set on a purposeful path of deterioration and ultimately death, perhaps enhancing survival of the species by making room for new individuals. Proponents of this view point out that different species of animals, despite their similarities at cellular and molecular levels, and, despite living in similar environments, nonetheless age at drastically different rates [2]. For example, mice live at most three to four years, twice the lifespan of the forest shrew, but only a tenth that of the naked mole rat, which has a lifespan of three decades. Therefore, the argument is that there must be some internally programmed clock dictating an organism to age and die. The other set of theories invoke the wear-and-tear concept, i.e., that aging is an accumulation of damage that causes cellular and organismal functions to decline and ultimately lead to death. A less-than-elegant way of describing this is that things simply “fall apart”, as entropy drives biological systems away from the intricate order essential for life. 

There are numerous problems with these theories, at least as they are presented above. In the case of programmed aging theories, several considerations raise concerns. First, when thinking about human aging in particular, only in the past two to three centuries out of the 200,000 years of human existence did aging become a major roadblock to the survival of most individuals. As Michel de Montaigne wrote in the 16th century:

To die of old age is a death rare, extraordinary, and singular, and therefore so much less natural than the others: it is the last and most extreme sort of dying […] And therefore my opinion is that when once forty years old, we should consider it as an age to which very few arrive […] and since we have exceeded the ordinary bounds which make the just measure of life, we ought not to expect to go much further [3].

With an average life expectancy of 30 years, it seems hardly likely that evolution, with its selection based on reproductive fitness, would have prioritized a program that only initiates well past expected lifespan. Second, and for the same reason (that evolution selects for reproductive fitness), a deleterious, pro-aging program that manifests after genes have already been passed to offspring is unlikely to be strongly selected against. Rather, it is likely that it is the decline in selective pressure that occurs with each additional offspring produced that allows for genetic mechanisms that contribute to aging to emerge and persist in the gene pool over evolutionary time [2]. A third, and more fundamental, problem with programmed aging theories is that they rely on circular logic: there is no need for an aging program to eliminate older individuals if there is no aging in the first place. Fourth, rare individuals in whom aging programs have been lost due to random mutation would, if anything, have greater reproductive success than their aging counterparts, and thus the program would tend to be lost gradually from the population over generations. Therefore, it is quite unlikely that aging is programmed per se, i.e., that its purpose is to ensure the decline and eventual death of individuals. Of course, this still leaves open the possibility that aging is driven by biological programs selected by evolution for other purposes. As described further below, a good example of such a program is cellular senescence, which helps prevent cancer into reproductive age, but also has the side effect of driving tissue aging. In addition, biological programs that do not drive aging, and may even protect against it, can modulate precisely how aging unfolds. Overall, although not programmed as an end in itself, aging involves highly mechanistic, and thus understandable and malleable, biological processes.

The wear-and-tear theories also leave unanswered questions, although it is fairly clear that in some fashion molecular and cellular damage are fundamental drivers of aging. In particular, the identities of the chief sources of damage remain uncertain, with current leading candidates including the damage and mutation of nuclear and mitochondrial DNA (including telomeres), oxidative damage, glycation, protein aggregation, immune dysregulation, and, as discussed below, the dysfunction of epigenetic mechanisms (each of these are reviewed in detail by [4,5,6,7,8,9,10]). Furthermore, these mechanisms, which involve seemingly random molecular damage, may not comport with our conceptions of how biological processes are impacted by aging. On the one hand, different individuals do experience aging distinctly, consistent with roles for stochastic damage. For example, some individuals are spared wrinkled skin but are profoundly affected by Alzheimer’s disease, whereas others succumb to cancer or cardiovascular disease despite many having lived highly health-conscious lifestyles. On the other hand, the fact that aging has highly stereotyped features, particularly within a species, is inescapable: people generally have no difficulty in distinguishing old individuals from young, and there are indeed many biological changes and hallmarks that occur with regularity, some even across different organisms [11].

There must, then, be ways in which the various types of molecular damage are summed into stereotyped biological outputs. Good examples of such integrators are the cellular stress responses, including a specialized form called cellular senescence. Cellular stress responses generally contribute to cell, and thus tissue and organismal survival in the face of various sources of damage and stress, including starvation, extremes in temperature, chemical toxins, and ionizing radiation. Examples of such stress responses are the use of metabolic flexibility to utilize available energy sources, activation of enzymes that neutralize oxidants and other toxins, induction of heat shock proteins that chaperone refolding of denatured proteins, upregulation of autophagy and other proteolytic mechanisms that can degrade damaged or aggregated proteins as well as provide new sources of cellular fuel, and the recognition and repair of DNA damage. Remarkably, genetic, dietary, or pharmacologic manipulations that promote longevity typically also enhance stress responses generally, supporting roles for these responses in countering aging, and indeed in many cases particular stress responses are known to be required for the enhanced lifespan and health span provided by various experimental manipulations [12,13,14]. Failures of these responses to fully resist or repair damage may therefore be key drivers of aging, and thus the weakest links in the web of stress responses may correspond to the most pro-aging insults. Because aging per se is not selected evolutionarily, but rather is due to the decline in selective pressure after genes have been passed to offspring, it should be freer to vary than many biological mechanisms, and therefore the particular links that are weakest may be different among species and even among individuals within a species. In this way, stress responses can help unify how cells experience damage while still leaving room for some variation in aging phenotypes.

Cell senescence is a programmed stress response different from those just described in that, rather than countering aging, it can contribute to it. It is activated by stresses that put cells at risk for becoming cancerous, including DNA damage, telomere dysfunction, chromatin perturbations, and mitochondrial dysfunction. Cell senescence is characterized by both a permanent arrest of cell division but maintained cellular viability, and by profound changes in cellular gene expression and physiology, including the secretion of inflammatory cytokines and proteases collectively called the SASP (senescence-associated secretory phenotype [15]). Although cell senescence protects younger individuals from cancer, the accumulation of senescent cells with age can interfere with normal tissue homeostasis. There is strong evidence that cell senescence can drive age-related pathology in two ways: (1) by limiting stem cell replicative capacity [16,17,18] and (2) by disrupting the tissues in which senescent cells reside. Of particular note, Jan van Deursen’s group showed that elimination of senescent cells forestalled the development of age-related pathologies in mutant mice with premature aging symptoms [19]. Recently, the same group showed that targeting and clearance of senescent cells occurring with natural aging in mice delayed tumorigenesis and slowed age-related deterioration [20]. Other studies have shown similarly that targeted apoptosis of senescent cells can restore fitness, hair density, and renal function [21], inhibit atherosclerosis [22], and ameliorate pulmonary fibrosis [23].

In summary, apparently stochastic forms of damage can activate programs that counter as well as contribute to aging, and in doing so unify cellular outputs and thus aging phenotypes caused by these diverse stresses. What types of damage are most important in driving aging is still in question. Given the central role of DNA as a repository of biological information, DNA mutations or other types of genetic damage have long been considered as candidates for key drivers. Although there is a strong body of circumstantial evidence supporting this hypothesis, it has been difficult to prove because we do not yet know of robust ways to selectively enhance DNA repair [24]. An alternative or complementary idea is that epigenetic damage, particularly to chromatin, contributes to aging by compromising the ability of the genome to code optimally for the maintenance of a youthful state. 

## 2. Epigenetic Regulation

When Conrad H. Waddington first coined the term “epigenetics” in the 1940s, the physical nature of genes was not yet known [25]. It would be another decade until Watson, Crick, Wilkins, and Franklin solved the structure of DNA and elucidated its essential role in information transfer in living materials. Waddington, a British developmental biologist as well as a left-wing politician, used the term epigenetics to describe the existence of mechanisms by which the environment can affect developmental processes. Fifty years later, Robin Holliday, a British molecular biologist and aging researcher, redefined epigenetics to encompass the study of changes in gene function that are heritable through mitotic and/or meiotic cell divisions and that do not involve any change in DNA sequence [26,27]. These definitions are not mutually exclusive, and there are indeed many cases of mechanisms that satisfy both definitions.

For nearly a century since Waddington first published the term, the field of epigenetics has grown exponentially as scientists search for explanations to differences in gene expression and function that cannot be attributed to changes in DNA sequence. Today, the term has expanded to include essentially any process that affects how gene-coded information is expressed to yield cellular and organismal phenotypes. These encompass mechanisms intimately connected with the functions of chromatin. Chromatin is a complex of DNA, histones, non-histone proteins and interacting RNA molecules. Its base unit is the nucleosome—comprising approximately 147 base pairs of DNA wrapped around eight core histones. Chromatin-based epigenetic mechanisms include methylation of cytosines within DNA, the actions of sequence-specific transcriptional activators and repressors, post-translational modification of histones, deposition by histone chaperones of variant forms of the core histones into nucleosomes, local repositioning of nucleosomes by chromatin remodelers, and the long-range organization of chromatin into loops, domains, and different degrees of compaction. Various non-coding RNAs also contribute directly to chromatin structure and function, and the regulation of messenger RNA (mRNA) stability and translation by small RNAs, including microRNAs (miRNAs) and small interfering RNAs (siRNAs), is also frequently considered to be a form of epigenetic regulation. Furthermore, epigenetic mechanisms help mediate the effects on gene expression of a plethora of environmental exposures and physiological states, including but not limited to, diet, hormonal changes with puberty and menopause, smoking, body mass, infection, time of day and season of the year, and even life experiences of past generations. An unfortunate side effect of this broad use of the word “epigenetics” is that it is now sometimes used by different people to mean substantially different things (reviewed by Deans et al. [28]). For example, geneticists lean towards Holliday’s definition and emphasize the heritability of gene expression patterns through generations of cells or organisms, whereas physiologists and ecologists tend to use the term to describe environmental effects on phenotypes, much like how Waddington first proposed. 

Imbued with all currently used meanings of the term, the biological territory thus encompassed by “epigenetics” includes essentially everything but unmodified DNA itself. It is no wonder, then, that epigenetic mechanisms should prove to be connected to aging in important ways. Nonetheless, it makes sense that, in particular, the chromatin-based mechanisms that underlie both Holliday and Waddington-type processes should be involved, from the views of both the wear-and-tear and programmed perspectives on aging.

First, from the wear-and-tear viewpoint, chromosomes are probably amongst the most information rich of all natural macromolecular structures, and all other things being equal, the greater the information content of a structure, the more susceptible it is to entropically driven decay. For example, the information content of the human haploid genomic DNA sequence is approximately 750 megabytes (two bits per base pair). The epigenetic information stored within a cell has not been calculated, but an estimate for the information potentially carried by the nucleosomes within a haploid genome, each with or without a mark at each of 64 different sites of histone posttranslational modifications (including phosphorylation, methylation, acetylation, or ubiquitination), was estimated to be at least 80 megabytes [29]. This is likely an underestimate, because there are in fact more than 64 modifiable positions, there are additional types of post-translational modifications (PTMs) e.g., sumoylation, neddylation, crotonylation, etc.), and some histone side chains can be modified in more than one way [30]. When one also considers the information potentially provided by the other dimensions of chromatin structure (DNA secondary structures, cytosine methylation, histone variants, transcription factor occupancy, nucleosome positioning, chromatin looping, regulation by small RNA molecules, etc.), and their combinations amongst one another and with the underlying DNA sequence, it is clear that the information content of chromatin is very large. Propagation of such a detailed molecular structure through rounds of cell division, as well as maintenance of such a structure in post-mitotic cells, would likely be challenging. This pessimistic view is counterbalanced by the existence of cooperative mechanisms for the maintenance of chromatin structures [31]. However, beyond basic considerations several lines of evidence suggest such stabilizing mechanisms do not fully prevent age-related losses in the fidelity of chromatin-based information. These include empirical evidence for increased cell-to-cell and gene-to-gene variation in gene expression with age [32,33,34], as well as inappropriate gene expression within aging cells, (i.e., “transcriptional drift” [35]); direct evidence for increased variance with age in 5-methylcytosine levels at numerous individual CpG sites within human genomic DNA suggesting a loss of information content (i.e., increased Shannon Entropy) [36]; and even the very interesting possibility that contributions to aging of stochastic losses in chromatin patterns are an unfortunate side-effect of an adaptive mechanism for providing phenotypic variation among individuals that operates via group-selection at the evolutionary level [37]. Remarkably, there is some evidence that suppression of transcriptional drift can delay aging. In particular, pharmacologic inhibition of serotonergic signaling in *Caenorhabditis elegans* promotes longevity and also suppresses transcriptional drift, although it is not yet clear if one causes the other, and if so, whether drift is upstream of aging [38]. 

Second, from the viewpoint of programmed aging theory, the central role that chromatin plays in the control of gene expression makes it likely, a priori, that chromatin-based mechanisms should affect aging in some way. In addition, there is good reason for excitement, because the generally reversible nature of epigenetic mechanisms raises hope that epigenetic changes impacting aging can be restored to a more youthful state, and thus ameliorate age-related diseases. In this review, we will first give a brief overview of evidence that epigenetic mechanisms are central to aging, and will then focus on two cellular elements that are intimate components of chromatin-based epigenetic mechanisms impacting aging: the levels of histone proteins and the function of telomeres.

## 3. Evidence for Epigenetic Regulation of Aging

Recent studies have shown that epigenetic mechanisms play an important role in aging. For example, a series of studies on individuals conceived during the Dutch famine show that nutritional deprivation leading to epigenetic changes in the periconceptional environment can have long-lasting consequences that contribute to the onset later in life of chronic diseases such as cardiovascular diseases, obesity, type II diabetes and hypertension. Interestingly, exposure to malnutrition in early vs. mid-gestation resulted in dysfunction of different organ systems [39,40,41]. Epigenetic studies of the same cohort showed less DNA methylation of the imprinted *IGF2* (insulin-like growth factor 2) gene in subjects prenatally exposed to famine compared to their non-exposed same sex siblings [42], but no significant differences in leukocyte telomere lengths were found [43]. A rodent model of periconceptional malnutrition showed that conditions causing intrauterine growth restriction (IUGR) and leading to diabetes in adulthood are associated with diminished expression of the *PDX1* (pancreatic and duodenal homeobox 1) gene, encoding a transcription factor essential for normal pancreatic beta cell development. These changes are associated with altered chromatin modifications at *PDX1* that are generally associated with reduced gene expression including reduced histone acetylation, reduced trimethylation of lysine 4 within histone H3, and elevated DNA methylation. Furthermore, these chromatin marks are reversed after treatment of the newborn rats with a peptide that counters the propensity toward diabetes, consistent with a role for these chromatin modifications in *PDX1* expression and thus beta cell failure [44,45].

Identical twin studies also provide clues about epigenetics and aging. A study of epigenetic markers in 80 pairs of monozygotic Spanish twins using peripheral blood lymphocytes, buccal mucosal epithelial cells and skeletal muscle biopsies showed that while epigenetically indistinguishable at earlier stages of life, markers such as total DNA methylation and histone acetylation levels began to differ as the subjects aged, a phenomenon known as “epigenetic drift”. Differences in gene expression patterns were four times greater in older twin pairs compared to younger ones [46]. Another study including nearly 2000 Danish twins measured the correlations of perceived age, rated from facial photographs, with aging phenotypes. A significant correlation was found between appearing aged and having short telomeres [47]. However, these studies are mostly observational, and do not provide evidence that the epigenetic differences observed are drivers, as opposed to passive correlates, of different aging phenotypes and longevity. Furthermore, although apparent differences in aging rate between genetically identical twins indicates that epigenetic factors must in some fashion be responsible, they do not reveal the nature of these factors.

Honeybees (*Apis mellifera*) offer interesting, and perhaps more convincing, insights into aging and epigenetics-regulated longevity. The fertile queen and sterile worker bees share identical genomic DNA sequences, but the queen bee has a lifespan 20 times that of the average worker, providing a striking example of the plasticity of longevity based on epigenetic mechanisms. Early studies of caste differentiation revealed the DNA methyltransferase (DNMT) Dnmt3 as an important factor. Downregulation of Dnmt3 by siRNA in newly hatched larvae resulted in reduced levels of methylation and increased the emergence of queen bees [48,49]. A genome-wide methylome study showed that thousands of genes display differential patterns of methylation in queen and worker larvae [50]. Fascinatingly, the lifespan of workers was increased significantly when they experienced pharmacological genome demethylation in early life by a topically applied drug called RG108, an inhibitor of DNMT activity. Further investigation showed that demethylation resulted in increased transcript and protein levels of vitellogenin, a protein known to be involved in honeybee caste differentiation and the regulation of longevity, though the effects were found to be indirect [51]. In addition, since the vitellogenin gene is naturally hypomethylated, further studies are needed to understand the nature of its regulation by DNMT activities. Although these findings are intriguing, more evidence is needed to ascertain the full importance and mechanistic basis for how DNA methylation changes modulate aging in honeybees.

In the past decade, the idea of a mammalian “epigenetic clock” based on changes in 5-methycytosine levels within genomic DNA has taken hold. A study of the human methylome, particularly at CpG sites, using whole blood found strong correlations between the genome-wide distributions of methylation and chronological age [36], while another study linked methylation-predicted age to higher mortality risk [52], suggesting it may be good marker of physiologic age, or at least of disease risk. In 2013, biomathematician Steve Horvath developed a multi-tissue predictor of age based on DNA methylation levels. Through years of perseverance and often solo work, Horvath collected and analyzed 8000 samples from 82 Illumina DNA methylation arrays datasets and discovered an accurate algorithm to predict chronological age (within 3.6 years) based on the methylation status of 353 CpGs. Since then, the epigenetic clock has been used to assess the effects of diet, exercise, education and other lifestyle factors on this molecular measure of aging. However, it is worth noting that DNA methylation, though an accurate biomarker of aging, is just one example of an epigenetic marker that can change in a meaningful way with age. For example, the global level and genomic distribution of histone methylation and acetylation patterns have also been reported to change with age (reviewed in [53,54,55]), and levels of histone acetylation were seen to vary more dramatically in older twins compared to younger twins [46]. Therefore, it is probable that all epigenetic markers indicate to some extent the rate of molecular aging, and together form an accurate model of physiologic aging. Again, matters of correlation and causality should be considered with caution, and much more work is needed to determine whether the “epigenetic clock” is a driver or simply a marker of age. The former possibility could afford us with exciting tools to manipulate molecular aging, though the actual execution would probably be very difficult, given the fact that epigenetic markers are (1) often widespread in the genome, (2) can be tissue- and cell-specific, and (3) often work in tandem with other marks as an “epigenetic code”. 

One of the most direct tests of the importance of chromatin in aging has come from genetic manipulation of epigenetic regulators such as histone and DNA methyltransferases, acetyltransferases, and chromatin remodelers. For example, deletion of the gene encoding the Sas2 acetyltransferase, which acetylates histone H4 lysine 16 (H4K16ac), delays aging in two different yeast aging models, the replicative aging of mother cells and the replicative senescence of telomerase-deficient mutants [56,57,58]. Furthermore, the ability to selectively mutate individual histone residues in yeast enabled confirmation that Sas2 regulates aging *via* H4K16, rather than other potential targets of its acetyltransferase activity. The H3K4me3 modification, often thought to be a marker of active gene expression, is intimately involved in aging across model organisms. In *C. elegans*, deletion of the genes encoding any of the three Trithorax group proteins that facilitate H3K4 trimethylation (WDR-5, SET-2 and ASH-2) results in decreased global levels of H3K4me3 and increased lifespan [59]. Similarly, in flies, deletion or RNA interference (RNAi) knockdown of the histone demethylase *Lid* resulted in increased levels of H3K4me3 and 15–24% reduced lifespan [60]. Remarkably, in the context of neurodegeneration driven by artificial expression in flies of the TDP-43 protein, which in humans is a key driver of amyotrophic lateral sclerosis and frontotemporal dementia, H3K4me3 appears to instead play a salutary role, promoting the recruitment of the Chd1 chromatin remodeler [58]. In addition, H3K4me3 is seen to redistribute within genomic chromatin of cultured human fibroblasts with senescence or following DNA damage [61]. H3K4me3 likely contributes to the transcriptional changes observed with aging, senescence and damage, although the exact mechanisms by which it modulates lifespan are still unclear. Similarly, H3K27 methylation and acetylation also contribute to lifespan regulation, and like H3K4me3 also is context-dependent, in this case operating quite differently in different species. In flies, loss of function mutations in genes encoding components of Polycomb Repressive Complex 2 (PRC2)—an H3K27me3 specific methyltransferase complex—result in decreased levels of H3K27me3 and increased lifespan [62]. Genetic manipulation of methyl- and acetyltransferases that elevate H3K27me3 and suppress H3K27ac offset the observed increase in longevity. In worms, however, the opposite effects are observed—increased levels of H3K27me3 through knockdown of its demethylase UTX-1 is associated with increased lifespan in an insulin dependent manner [63]. Apart from histone posttranslational modifications, other types of chromatin alterations also impact lifespan. For example, overexpression of the DNMT2 DNA methyltransferase increases lifespan in *Drosophila* [64], whereas deletion of *ISW2*, which encodes a chromatin remodeler, delays both the aging of yeast mother cells and the senescence of yeast telomerase mutants [65]. Remarkably, stress resistance in general is enhanced by many of the above longevity-promoting mutations in chromatin regulators, indicating that regulation of stress response genes may be a key mechanism by which chromatin counters aging. An alternative, and fundamentally different interpretation is that disruption of normal chromatin regulation creates mild and essentially non-specific stresses that activate stress responses in a tonic fashion so as to make organisms more resilient in the face of potentially more deleterious age-related stresses, so-called “stress hormesis” [66]. It will be important in future studies to establish the relative contributions of these two ways in which chromatin-based regulation of stress resistance may contribute to longevity.

Ground-breaking work culminating in the cloning of the sheep Dolly demonstrated that cell nuclei taken from highly differentiated adult tissues and introduced into an enucleated oocyte can be rejuvenated and reprogrammed into pluripotent stem cells that give rise to a younger version of the donor animal. In particular, the nucleus used to generate Dolly was obtained from the mammary gland of a six-year-old sheep, and thus whatever nuclear changes drive aging are apparently reversed by the cloning process. However, due to Dolly’s early onset of osteoarthritis, premature euthanization, and apparently diminished telomeres [67], there has long been concern that Dolly might not have started from “age zero”. Recent evidence from radiographic examinations of Dolly’s skeleton suggests, however, that the amount of arthritis she suffered was comparable to that of an age-matched naturally conceived sheep, and that she was not abnormally aged physiologically for a sheep of her chronological age [68]. Moreover, Dolly’s sisters, clones derived from the same mammary gland cell line that gave rise to Dolly, have cruised into old age for sheep (7 and 9 years, with the typical sheep lifespan <10 years) and are relatively healthy in their sunset years [69], suggesting that cloning is indeed one way to rejuvenate old somatic cells. 

Even more exciting, recent work has demonstrated that rejuvenation is possible in adult animals through partial reprogramming of the epigenome. In 2006, induced pluripotent stem cells were first generated by ectopic expression of what became known as the Yamanaka factors (Oct4, Sox2, Klf4, c-Myc) [70,71,72]. Yamanaka factors are believed to initiate most developmental signaling pathways, and their transient expression in cultured cells can induce the cells to return to a de-differentiated state [73,74]. Short exposures to Yamanaka factors in culture can result in partial reprogramming and the suppression of aging hallmarks (senescence-associated β-galactosidase activity, reactive oxygen species and expression of aging-associated stress response genes), without complete loss of cellular identity. Recently, Yamanaka factors were transiently expressed in vivo in a mutant *LMNA* mouse model of the Hutchinson-Gilford premature aging syndrome, and astoundingly, resulted in an increase in median and maximal lifespan, and alleviated aging phenotypes in cells and in multiple organs, including markers of DNA damage, senescence, and age-related changes in H3K9me3 and H4K20me3 [75]. Furthermore, in naturally aged mice, transient expression of Yamanaka factors diminished several molecular markers of aging and also enhanced recovery following acute pancreatic and skeletal muscle injuries. These findings emphasize the importance of epigenetic programs as drivers of aging, and demonstrate that they can be reset to more youthful states even in later stages of life. 

Besides those mentioned above, how epigenetic mechanisms regulate aging, including roles for caloric restriction and metabolic regulation, have been extensively reviewed elsewhere [55,76,77,78,79]. Below we focus specifically on how histone levels and telomeres, and some of their interconnections, influence aging processes. 

## 4. Aging-Related Histone Losses

Loss of histone proteins appears to be a conserved feature of aging from yeast to humans, and is of functional importance in at least some of these cases. Yeast mother cell aging is accompanied by a profound loss of total cellular histones, including a ~50% decrease in nucleosome occupancy genome wide, and is accompanied by less defined positioning of remaining nucleosomes to their usual sites within chromosomes [80,81]. Similar histone losses occur with the replicative senescence of telomerase-deficient yeast, and this is clearly of major importance for determining the altered gene expression in senescent cells because these patterns are closely mimicked by the artificial downregulation of histones [82,83]. Moreover, artificial overexpression of core histones promotes longevity in both yeast models of aging, indicating that histone losses may be a key driver of aging. Similar age-related histone losses have been reported in *C. elegans* worms [63]. Furthermore, genetic inactivation of the SET-26 histone methyltransferase or lithium treatment enhance both lifespan and levels of histones, or mRNAs encoding histones, respectively [84,85]. These observations suggest that such histone losses may limit longevity in worms, although because each of these manipulations affect more than histone expression (e.g., SET-26 affects H3K9 methylation and lithium has widespread metabolic effects) it is possible that other effects of these manipulations explain the increased lifespan.

Similar decreases in histone synthesis and global histone levels have been observed in mice and humans, although the functional importance of such changes is not yet clear. A decline in histone mRNAs in quiescent (i.e., non-cycling) mouse skeletal muscle stem cells occurs with age, which might contribute to known age-related losses in function of these cells, though it is notable that these transcripts return to normal levels when the stem cells are activated by acute injury [86]. Cultured early-passage fibroblasts from a 92-year-old were found to have 50% less synthesis of histones compared to those from a nine-year-old [87]. Furthermore, human IMR90 and WI38 fibroblasts passaged extensively in culture to drive their replicative senescence demonstrated significant changes in histone biosynthesis and processing, including downregulation of histone H3 and H4 synthesis and extensive changes in the levels of several histone PTMs [87]. In vivo histone loss in human skin was also observed in the melanocytes of benign (i.e., pre-malignant) nevi. Interestingly, depletion of histones was most marked in regions of the nevi that have been inversely linked with malignancy, suggesting that histone loss is linked to enhanced senescence-associated proliferation arrest and thus tumor suppression [88].

**Figure 1:**
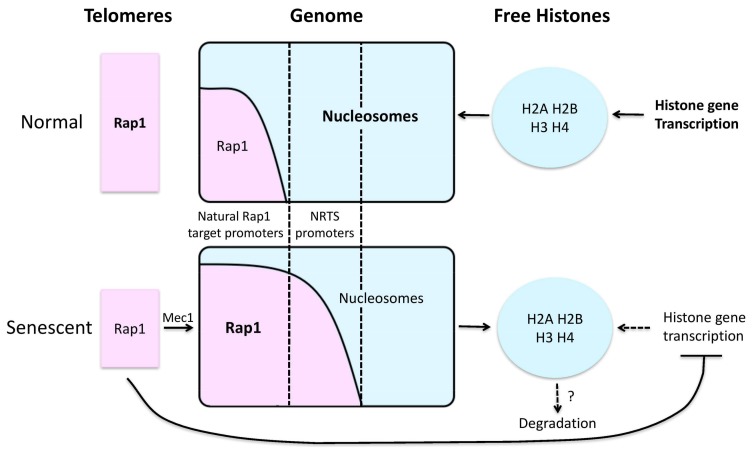
Rap1 relocalization at senescence in telomerase-deficient yeast leads to global and site-specific histone losses. Shown are the relative levels of Rap1 and histone proteins at telomeres, nontelomeric genomic regions, and in non-chromatin-bound histone pools. In normal cells, Rap1 is clustered at telomeres and is present at the promoters of natural Rap1 target genes. When cells become senescent due to critically short telomeres, Rap1 relocalizes to the promoters of natural and new Rap1 targets at senescence (NRTS). This relocalization depends on the Mec1 checkpoint kinase, which is activated by uncapped telomeres. At NRTS promoters, Rap1 binding displaces histones, resulting in site-specific histone losses. Rap1 also relocalizes to the promoters of histone genes and inhibits their transcription. Thus, the increased availability of Rap1 together with a decline in histone levels contribute to increased Rap1 occupancy at NRTS at senescence. Global histone levels also decrease in response to non-telomeric DNA damage, mediated by histone degradation driven by the Rad53 checkpoint kinase, but whether such degradation occurs at senescence, and whether Rap1 is involved in generic DNA damage responses have not been tested (see text).

How histone levels decline, and how such declines drive aging, are poorly understood. In the case of human senescent fibroblasts, histone losses were suppressed by artificial expression of telomerase, and therefore DNA damage signals coming from shortened telomeres are likely to be involved [87]. In the case of senescence in yeast telomerase mutants, a key role is played by Rap1 [82]. Rap1 is a telomere DNA binding protein and sequence-specific transcription factor that typically activates transcription, and does so at roughly 5–10% of all yeast genes, particularly the highly expressed genes encoding ribosomal proteins and glycolytic enzymes. In certain contexts, Rap1 can instead function as a repressor, notably at telomeres and the silent mating loci. There is longstanding evidence that although Rap1 can bind to nucleosomal DNA, it can also help exclude nucleosomes from sequences around its binding sites, thus contributing to transcriptional activation [89,90,91,92,93]. In senescent cells Rap1 relocalizes from shortening telomeres and subtelomeres to the promoters of hundreds of new genes [82]. These include those encoding the core histone proteins, which are transcriptionally repressed by Rap1, thus contributing to diminished global histone levels. However, most new targets of Rap1 in senescent cells are upregulated by Rap1, and this is accompanied by a preferential loss of histones at the promoters of these particular targets. Thus, global and site-specific inhibition of histones appears to contribute to transcriptional activation by Rap1 at senescence (Figure 1). Although levels of Rap1 at its normal target promoters are maintained or increased at senescence, transcripts from these genes decrease in comparison to total levels of transcripts. Based on this, and the consideration that because normal Rap1 target promoters are naturally nucleosome poor they are unlikely to be further upregulated by global losses in histones, it was proposed that global nucleosome losses lead to a general upregulation of most genes in senescent cells [82]. Indeed, through the use of careful controls in a different yeast aging model it was demonstrated that transcription of essentially the entire genome is indeed elevated in old yeast mother cells [81]. In both yeast mother cell aging and IMR90 senescence, the total synthesis of histones is downregulated, but underlying mechanisms are not known. Besides reduced synthesis, increased degradation of histones may also be involved. For example, senescent cells show an upregulation in autophagic and lysosomal activities, and inhibition of the lysosomal pathway antagonized the decrease in histone content in senescing IMR90s [88]. Furthermore, chromatin in senescent cells is ubiquitinated and co-localized with so-called p62 bodies, which recognize and target ubiquitinated proteins for autophagic degradation [88]. Despite this evidence for autophagy driving aspects of cellular senescence, several lines of evidence indicate that autophagy actually helps promote longevity [94,95,96,97,98,99], and thus any role for autophagy in age-related histone losses seem unlikely to be a major determinant of its effects on aging overall. Interconnections between histones and autophagy during aging were also revealed by the combined demonstrations that (1) nucleocytosolic depletion of acetyl-CoA enhanced autophagy and extended chronological lifespan in yeast; and (2) locking two histone residues that display age-related hyperacetylation (H3K14 and H3K18) into an effectively intermediate state of acetylation increased age-related autophagy [100,101]. However, more work is needed to address the precise mechanisms that link histone levels and PTMs to autophagy pathways. 

If histone losses are an important driver of aging, how exactly they contribute is still an open question. However, it is likely that inappropriate gene expression is involved. For example, a decline in global histone levels might contribute to heterochromatin losses, which in turn could lead to derepression of genes and aberrant gene expression. In worms, age-dependent loss of peripheral heterochromatin and changes in nuclear architecture were detected in non-neuronal cells [102]. In *Drosophila*, decreased heterochromatin correlates with shortened lifespans and vice versa [103]. In humans, cells cultured from HGPS patients showed a loss of heterochromatin along with loss of heterochromatic marks H3K27me3 and H3K9me3 [104]. Skin fibroblasts from normally-aged individuals showed similar defects compared to HGPS patients, including nuclear aberrations, epigenetic changes and accumulation of DNA damage [105]. ATAC-seq analyses of human peripheral blood mononuclear cells revealed age-related stochastic opening of silenced genomic regions, but along with decreased chromatin accessibility in regulatory regions associated with T cell signaling [106]. In addition, a decline in total histone levels may lead to preferential losses at particular genomic regions, and thus an imbalance in the expression of genes (transcriptional drift), which may compromise the ability of cells to perform normal functions and respond to stresses. How the distribution of nucleosomes throughout the genome changes with age is understudied, but careful measurements in aged yeast mother cells indicate that remaining nucleosomes mostly become delocalized [81]. However, this view comes from examining populations of old cells, and thus whether nucleosomes might linger at different particular loci in individual cells remains unclear. An alternative way in which preferential nucleosome losses at particular genomic regions might contribute to aging is toxicity from the overexpression of certain genes. Along these lines, there is a growing body of evidence that depression of transposable elements, which normally silenced in healthy cells, occurs with aging and may also drive age-related defects. For example, age-dependent increased expression of long terminal repeat (LTR) retrotransposons have been observed in the *Drosophila* brain [107]; retrotransposon elements (RTEs) and satellite sequences increased with age in both in vivo mouse models and cultured human diploid fibroblasts [108]; and long interspersed nuclear elements (LINE), short interspersed nuclear elements (SINE) and LTR transposon derepression is commonly seen in neurodegenerative disorders in humans [109,110,111,112] . 

Another level of chromatin dynamics includes the exchange of canonical histones with histone variants. Histone variants are isoforms of canonical histones that are generally expressed throughout the cell cycle and can be incorporated into chromatin in ways that are replication-independent and highly dynamic. Their substitution for canonical histones affects chromatin organization and can thereby regulate important cellular processes. A growing body of evidence links histone variants to aging. For example, a study of the H2A and H3 histone families in cultured human fibroblasts show that histone variant amount and biosynthesis levels change with aging, with upregulation of H2A.2 and H3.3 as well as downregulation of H2A.1 and H3.1 [113]. Consistently, H3.3 accumulation was observed with development and aging. For example, mass spectrometry performed on mouse somatic tissues at various ages (3–24 months) revealed an accumulation of H3.3, with near complete replacement of canonical H3.1/H3.2 by H3.3 by the age of 18 months [114]. In mouse neuronal chromatin, H3.3 constituted only a small fraction of the histone H3 pool in embryonic stages, whereas it accumulated to nearly 94% of the H3 pool by old age (2 years). Similar levels of accumulation were found in postmortem human brain, starting from 31% in fetal brain and gradually increasing to >93% through the first decade of life [115]. Despite these accumulations, the turnover of H3.3 in neurons remains highly dynamic and contributes to gene regulation in a fashion dependent on neuronal activity and proteasomal degradation, but apparently independent of activating post-translational modifications [115]. H3.3 has traditionally been associated with active chromatin, and though it differs from its canonical counterparts only at a few amino acids, it utilizes different histone chaperones for incorporation, namely HIRA and DAXX/ATRX [116]. Its accumulation with age and apparent high turnover rates compared to canonical histones raises the possibility that H3.3 incorporation is another way in which aged chromatin may become more “open” and flexible. 

Histone variant H2A.J is another histone H2A variant that has been linked to aging. H2A.J differs from the canonical H2A by only five amino acids and is only found in mammals. Recent studies have reported that H2A.J accumulates in senescent human fibroblasts with persistent DNA damage. It was also found to accumulate in mice in an age- and tissue-dependent manner. Overexpression of H2A.J increases the expression of inflammatory genes such as *IL1A*, *IL1B* and several interferon-inducible genes that contribute to the senescence-associated secretory phenotype (SASP), whereas H2A.J knock-down inhibits the expression of these genes [117]. SASP factors can affect surrounding cells through the alterations of cellular microenvironments and promote chronic inflammation and in some cases, aging-related diseases and cancer progression. H2A.J may offer a novel channel by which to manipulate the SASP, although further investigations are needed to reveal more about how H2A.J accumulates at senescence and regulates SASP gene expression.

Despite a chromatin opening effect of global losses of histones, senescent cells nonetheless form more compact foci of chromatin, termed senescence associated heterochromatic foci (SAHF) [118]. SAHFs contain several markers characteristic of heterochromatin, including hypoacetylated histones, heterochromatin protein 1 (HP1), the histone variant macroH2A, and H3K9 methylation. SAHF can result in heterochromatization of proliferation-promoting genes such as cyclin A, which may contribute to the stable proliferative arrest of senescent cells. Interestingly, DNA sequences typically found in constitutive heterochromatin, such as pericentromeres and telomeres, are excluded from the bulk of SAHF, consistent with a general loss of heterochromatin and expression of RNA from these elements in senescent cells [118,119,120,121,122]. The story of how SAHFs perturb chromosome architecture and regulate gene expression is quite complex and far from complete. For example, SAHFs are not formed in all senescent cell nuclei [123,124]. In addition, SAHFs are composed of multiple types of chromatin and arranged in layers with an H3K9me3 enriched constitutive heterochromatic core encircled by an H3K27me3 enriched facultative heterochromatic outer ring [125]. It is not surprising that compartmentalization of constitutive heterochromatin, facultative heterochromatin, and euchromatin can regulate gene expression, though the details of how this is achieved are still unknown. 

The DNA damage response (DDR) is likely to be one important trigger of histone losses with age. Senescing human fibroblasts accumulate DNA damage at telomeres and elsewhere, and thus activate the DDR [126], and it was proposed that DDR-mediated decreases in levels of the stem-loop binding protein might contribute to diminished expression of histones [87]. Histone losses driven by Rap1 in senescing yeast require Mec1, an ATM (ataxia-telangiectasia mutated) and ATR (ATM- and Rad3-related) homologue that mediates much of the DDR, including that activated in response to critical telomere shortening [82]. Because critically shortened telomeres resist repair, it was proposed that, like other difficult to repair DNA breaks, the purpose of induced histone losses might be to enhance both chromatin flexibility and “openness” so as to stimulate the search for homology and invasion of intact template DNA by broken ends that underlies repair by homologous recombination (HR) [82,127,128]. In other words, a mechanism that is helpful for the repair of non-telomeric DNA breaks in normal cells might also be activated in senescent cells though perhaps without benefit. Indeed, experimental depletion of histone H4 enhances HR [129], and, moreover, it has been found that cellular histone levels drop by 20–40% genome-wide in response to DNA damage [130], a degree comparable with senescent cells. This loss is proteasome-mediated and requires both the DNA damage checkpoint and the INO80 chromatin remodeler [130]. 

Several lines of evidence suggest that histone chaperones and chromatin remodelers also could be key players in reshaping the chromatin landscape during aging and senescence. Asf1, a histone H3/H4 chaperone that facilitates histone deposition, exchange and removal, and serves as a “histone buffer” to ensure that a pool of free histones is readily available to be deposited [131], has been linked to histone loss and chromosome condensation. In yeast mothers, cells lacking Asf1 are short-lived by about 20 generations compared to wildtype [80]. The mechanism by which this is achieved is still unclear, though it is possible that the presence of Asf1 facilitates dynamic exchange of damaged histones and increases histone deposition to repress stochastic gene transcription [132]. An alternative mechanism that might be of particular importance in the setting of critically shortened telomeres or other sources of DDR signaling, could involve regulation of the dynamics of histone levels. Because yeast Asf1 binds soluble histones, and moreover, through direct binding inhibits the ability of the DDR checkpoint kinase Rad53 to direct the proteolytic degradation of histone proteins [133,134], Asf1 might be important to restrain excessive histone degradation. Furthermore, histone H3 and Rad53 compete for binding to Asf1, and therefore the diminished levels of histones with age or senescence would free up Asf1 to inhibit Rad53, so that when the DDR ceases following repair of any reversible telomere or other DNA damage, Rad53 would be robustly and rapidly inhibited, leading to restoration of histone levels and resumption of the cell cycle [135]. Consistent with this idea, in late passage IMR90 fibroblasts, ASF1a and ASF1b were substantially downregulated [87]. Also of note, in cooperation with the chromatin regulator HIRA and involving direct binding to histone H3, ASF1a plays a key role in the formation of SAHF [122,136]. 

If losses of histones at particular genomic loci contribute to aging, then it stands to reason that adenosine triphosphate (ATP)-dependent chromatin remodelers, which can reposition nucleosomes away from some sites and toward others, might also regulate aging. Indeed, several such enzymes have been implicated in age-associated chromatin reshaping and lifespan regulation. There are at least five families of chromatin remodeling ATPases that are highly conserved: SWI/SNF, ISWI, CHD/NuRD, INO80, and SWR1 [137]. As mentioned above, it was found that in yeast, deletion or mutation of *ISW2*, encoding the catalytic component of the ISW2 complex, delays yeast mother cells’ aging and replicative senescence [65]. Further investigation suggests that deletion of *ISW2* and caloric restriction, which delays aging in many organisms, show significant overlap in nucleosome positioning shifts, suggesting that they may share a common pathway in promoting lifespan extension. In *C. elegans*, the subfamily SWI/SNF was linked to DAF16-mediated gene transcription, stress response and longevity [138], possibly through chromatin remodeling mechanisms. It is worth noting that deletion of *ISW2* in *Saccharomyces cerevisiae* and the action of SWI/SNF in *C. elegans* appear to trigger stress responses, adding weight to the idea chromatin manipulation may promote longevity by promoting stress resistance via stress hormesis.

## 5. Telomere Structure and Function

Telomeres are the structures at chromosome ends, and comprise tandem repeats of DNA, which in vertebrates have the sequence 5′–TTAGGG–3′, and end with a 3′ single stranded overhang at least 100 nucleotides in length. Vertebrate telomeres are usually “capped” by shelterin complex proteins, including TRF1, TRF2, RAP1, TIN2, TPP1, and POT1 [139]. For several reasons, telomere length can shorten with each cell division. This is often attributed to the so-called end replication problem, which is the inability during genome replication of the RNA that primes lagging strand synthesis of the final Okazaki fragment to be replaced by DNA. During replication of most of the genome, subsequent Okazaki fragments extend in the 3′ direction to replace the RNA primers priming the previous fragment, thus replacing it with DNA. However, because no such subsequent fragment exists at the chromosome end, the removal of this RNA primer leaves a gap that is not filled by DNA synthesis. This would make sense as a cause of telomere shortening save for the fact that telomeres naturally contain long 3′ overhangs, which means that, if the RNA primer is placed at the very chromosome end, no shortening of the lagging strand need occur. The actual reasons for telomere shortening caused by normal genome replication, at least in the human cells studied, appear to be that (1) rather than being placed at the very end of the telomeric DNA, the final RNA primer for lagging strand synthesis is placed internally and (2) after replication generates the blunt ended product of leading strand synthesis, it is exonucleolytically processed so as to develop the 3′ single stranded overhang necessary for telomere functions [140,141]. On top of these considerations, direct damage to telomere DNA (e.g., oxidative damage) and occasional replication fork collapse within telomeres, which are inherently difficult to replicate at least in part due to DNA secondary structures, also degrade telomeres and can sometimes lead to sudden and dramatic shortening [142,143,144,145,146,147,148]. Telomere shortening can be countered by the action of telomerase, which can synthesize new repeats at telomere ends. However, in humans telomerase activity is restricted primarily to the progenitor cells of high turnover tissues, and telomeres shorten with age in most tissues [149,150]. 

Telomeres occupy an interesting zone spanning the border between genetics and epigenetics. One the one hand, although telomeric DNA does not code for any known protein, the DNA sequences of telomeres nonetheless matter in a genetic sense, mainly for the binding of shelterin complexes. One the other hand, the length of the telomere repeats is also important for telomere functions, and this length is both heritable and can increase or decrease reversibly, similar to other epigenetic mechanisms. Telomere length has proven to be an important regulator of gene expression and cellular signaling. The most well-established mechanisms involve triggering of DDRs by critically shortened, i.e., “uncapped”, telomeres, which lead not only to gene expression changes but also to cellular senescence or apoptosis. Recently, it has also been shown that even before a telomere has become short enough to be uncapped, its shortening can influence gene expression, as discussed in detail below. In addition to regulation of gene expression by telomere length, telomeres, subtelomeres, shelterin proteins and telomerase are all themselves subject to epigenetic regulatory mechanisms [79,151]. Of particular note, in mice and zebrafish the mRNA levels from the genes encoding the six shelterin subunits change with age, in a tissue specific fashion [152], and it will be exciting to test if these changes may impact age-related pathologies. 

## 6. Contributions of Telomeres to Human Aging

Several lines of evidence indicate that changes to telomeres contribute significantly to human aging. Although much of this evidence has historically been indirect or correlational, recent lines of evidence are more convincing. We, and others, have reviewed this evidence in detail [10], and so will only summarize it here. First, it is clear that telomeres shorten in most human tissues with age [150]. Second, telomere shortening can lead to cell senescence or apoptosis in cultured cells [153]. Third, the progeroid syndromes dyskeratosis congenita, Werner syndrome, and Hutchinson–Gilford progeria syndrome include telomere defects, and several lines of evidence indicate that these contribute to premature onset of various age-related diseases affecting these individuals [154,155,156,157,158,159,160,161,162]. Fourth, up to 15% of dermal fibroblasts in elderly baboons contain uncapped telomeres and signs of cell senescence [163,164], although similar studies have not yet been reported in humans and in other tissues. Fifth, telomere shortening in easily measured cells (peripheral leukocytes and buccal epithelial cells) is correlated in cross-sectional epidemiological studies with age-related diseases, including cardiovascular diseases, diabetes mellitus, osteoporosis, pulmonary fibrosis, cirrhosis, and cancer [165,166,167,168,169,170,171]. Sixth, within diseased tissues telomeres are shortest at sites of pathology, including the vascular smooth muscle cells of atherosclerotic plaques and fibrotic areas of the lung [172,173].

None of the above observations demonstrates definitively that telomere shortening drives aging. For example, it could be argued that short telomeres are only a marker of disease, being caused by the same stresses that lead to the disease itself [174]. However, two lines of evidence more strongly support a causal role for telomere shortening in natural aging. First, overexpression of telomerase can extend mean lifespan and slow aspects of aging in mice [175,176]. This is true even though, compared to humans, mouse aging is relatively unaffected by telomere shortening, which can be seen by comparing the nearly normal health of first-generation mice fully lacking telomerase to people with dyskeratosis congenita, who only partially lack telomerase activity but nonetheless become sick, typically in adolescence or early adulthood. These considerations suggest that improved telomere maintenance may even have greater effects in humans, though we caution that this likely includes elevated cancer risk, especially in younger individuals. Second, and moreover, so-called Mendelian randomization studies in humans argue that short telomeres drive several age-related pathologies, including cardiovascular diseases, pulmonary fibrosis, diabetes mellitus, and even Alzheimer’s disease [177,178,179]. These studies quantify the extent to which different alleles affecting telomere length (for example in *TERT*, *TERC*, and *NAF1*, genes required for full telomerase activity) impact disease risk in proportion to their effects on telomere length. The demonstration, for example, that someone born with alleles that lead to telomere lengthening has a lower risk for cardiovascular disease, and that this lower risk applies to other similar alleles in proportion to their effects on telomere length, argues clearly that the longer telomeres are responsible for the lowered disease risk. Remarkably, such individuals are also at higher risk for various cancers, explaining why humans have not simply evolved longer telomeres or higher levels of telomerase, which from a purely technical standpoint is easily accomplished and in fact characteristic of many species. This lends weight to the hypothesis that human telomere length is an example of antagonistic pleiotropy: it reflects a compromise between deleterious effects later in life and protection against cancer earlier in life that supports fitness and fecundity. Thus, the set point for human telomere length reflects a tug-of-war between death from cancer and death from degenerative diseases. Together, these findings support a causal role for telomeres in age-related diseases.

## 7. Chromatin-Based Mechanisms by which Telomeres May Regulate Aging

As noted above, changes in telomere length, including those that lead to critical shortening, can perturb cell biology in a myriad of ways, leading to dramatic changes in gene expression and chromatin architecture, which in turn may contribute to cell and tissue dysfunction. We will briefly list these mechanisms, and then discuss them below. First, critically short telomeres can be recognized by the DNA damage response machinery as double strand breaks, triggering a series of downstream events. Second, telomeres bear heterochromatic marks that can repress nearby gene expression, a phenomenon known as the telomere position effect (TPE). Telomere shortening can result in loss of TPE and perturb gene expression patterns. Third, the telomere end is coated with proteins that can redistribute to extratelomeric loci either as a result of telomere shortening and loss of binding sites, or by being actively recruited by cellular machinery in response to various signaling pathways, such as the DDR. Fourth, telomeric DNA is transcribed into long non-coding RNAs called telomeric repeat-containing RNA (TERRA). TERRA is involved in maintaining telomere integrity and is known to interact with telomeric and extratelomeric DNA as well as some proteins to regulate gene expression. TERRA generally increases in abundance when telomeres become short, and may thus communicate telomere length changes with the wider genome. 

Telomeres can shorten to the point where they become uncapped and recognized by DDR factors as double-strand DNA breaks. For example, in mammals, these telomeric signaling events can activate the ATM kinase to signal to p53, causing upregulation of p21 and G1 phase arrest, and similar mechanisms are used in other organisms [126,180,181,182]. Persistent DDR signaling at short telomeres can lead to cellular senescence or apoptosis. In addition, telomere uncapping can activate the NF-κB signaling pathway. NF-κB is found in almost all animal cell types, and is an evolutionarily conserved response to internal and external stresses, such as oxidative and genotoxic damage. Inhibition of NF-κB reduces oxidative stress, oxidative DNA damage and senescence, both in cultured cells and mice [183]. A genome-wide screen of regulators of the NF-κB pathway found the shelterin protein Rap1 as one of its targets. Further investigation revealed that ectopic expression of Rap1 induces NF-κB, whereas depletion of Rap1 inhibits NF-κB activity [184]. Mechanistically, Rap1 can enhance the recruitment of p65—an IκB kinase (IKK) substrate—through association with members of the IKK complex, which is essential for Rap1-mediated activation of NF-κB regulated genes. Furthermore, levels of Rap1 are positively regulated by NF-κB. This suggests that telomere shortening and thus possibly the release of Rap1 from telomeres might contribute to the activation of NF-κB during aging. 

Additionally, even before a telomere has become short enough to be uncapped, its shortening can influence gene expression. It has long been known that chromatin at telomeres bears heterochromatic marks that can “spread” into and silence nearby genes, a phenomenon known as the “telomere position effect” (TPE). TPE was first described in yeast, and in general the repressive effects of TPE are in proportion to telomere length [185]. In yeast that senesce due to genetic inactivation of telomerase, telomere shortening was shown to be accompanied by an increase within subtelomeric regions of the acetylation of H4K16, a euchromatic mark [57]. Acetylation was accomplished by the Sas2 acetyltransferase, and deletion of *SAS2* or mutation of H4 lysine 16 delayed senescence, apparently by release of the Sir3 heterochromatin protein from telomeres, thus improving telomere repair by homologous recombination. TPE in human cells was first described in 2001 by Baur et al. when it was discovered that similar to subtelomeric gene repression in yeast, reporter genes integrated close to a telomeres were expressed at a 10-fold lower abundance compared to controls generated by random integration [186]. Subsequently, the same group showed that TPE could be lost with telomere shortening [187]. Apart from simple loss of telomeric heterochromatin due to attrition, telomere shortening in mice can also lead to a series of epigenetic changes at telomeres and subtelomeres, including a decrease in the density of heterochromatic marks (H3K9 and H4K20 trimethylation, HP1 binding) and an increase in “open” chromatin indicated by H3 and H4 acetylation [188]. This hyperacetylation is in line with observations that telomeres are excluded from the bulk of SAHF formation in senescent cells, and it may contribute to losses in transcriptional silencing at subtelomeric regions. Furthermore, aside from repressing nearby subtelomeric genes, telomeres at the end of long chromosome loops in mammalian cells can be brought into close proximity to genes up to 10 Mb away. This spatial proximity apparently allows the heterochromatic telomere effects to spread into the affected genes, repressing gene expression. As telomeres shorten, the chromosome loop diminishes, thus alleviating gene repression [189]. Remarkably, the human telomerase reverse transcriptase (hTERT) locus, encoding the catalytic subunit of telomerase, is regulated by this mechanism, which might contribute to telomere length homeostasis [190]. Therefore, telomere shortening as a result of replicative senescence leading to loss of TPE at subtelomeric regions and also over long distances can alter gene expression and protein levels, though it is still unclear how it contributes to natural aging and age-related pathologies. However, connected with this idea, telomere length and TPE was found to contribute to the pathogenesis of facioscapulohumeral muscular dystrophy (FSHD). FSHD is one of the most frequent hereditary muscle disorders characterized by progressive muscle weakening, with the age of onset typically around teenage years. Loss of repression of DUX4, a transcription factor genetically only 25–60 kb away from the end of chromosome 4q, is one of the primary reasons of FSHD pathogenesis. Not unexpectedly, DUX4 expression was shown to be inversely correlated to telomere length [191], suggesting that loss of TPE with age is at least one of the mechanisms that contribute to FSHD.

Another way in which telomeres regulate gene expression is the redistribution of proteins typically found at telomeres to new genomic loci. This could be due to loss of their high-affinity telomeric binding sites in cases of telomere shortening, or active dissociation from telomeres even without shortening through the triggering of other related pathways, e.g., the DNA damage response. For example, it has long been known that the SIR silencing proteins, recruited to telomeres via Rap1, relocalize to the nucleolus in old yeast mother cells [192], where they promote survival and extend lifespan. Consistently, increased expression of SIRT1—the mammalian ortholog of Sir2—in a mouse model of genomic instability suppresses age-dependent gene expression changes, and promotes lifespan [193]. Recently, it has been reported that TRF2 can dissociate from telomeres and redistribute throughout the nucleoplasm in primary and human cancer cells in response to heat shock [194]. In addition, the yeast homologue of the shelterin protein RAP1 was found to relocalize to the promoters of new Rap1 targets at senescence (NRTS) as a consequence of telomere shortening in the telomerase deficient yeast model [82]. A direct result of Rap1’s presence at NRTS promoters is their upregulated expression. Another interesting outcome of Rap1 relocalization is genome-wide and site-specific histone losses. In detail, Rap1 was found to bind at the promoters of all core histone genes and represses their expression, contributing to a global loss of histone levels. In addition, Rap1 also contributes to local nucleosome losses at activated NRTS genes, possibly through recruitment of histone eviction machinery (Figure 1) [82]. Interestingly, mouse Rap1 was also found to relocalize with progressive telomere shortening, resulting in transcriptional changes [195]. However, because these experiments were performed in immortalized mouse embryonic fibroblasts (MEFs), it is unknown whether Rap1 redistribution in mammals drives histone losses and other aspects of cellular senescence as it does in yeast, although the connections between Rap1 and NF-kB described above support such a potential role. 

TERRA is a telomeric repeat-containing long non-coding RNA transcribed by RNA polymerase II from telomeres, and plays an important role in telomere maintenance and function [196]. TERRA transcription has been observed across multiple organisms, including yeast, zebrafish, mouse and human. TERRA molecules can associate with telomeres to form a DNA:RNA hybrid D-loop known as an R-loop. R-loops have been observed in both yeast and humans. In yeast, TERRA was found to accumulate as a result of telomere shortening, clustering onto the short telomere from which it was transcribed, and serving as a nucleation site for telomerase recruitment and subsequent telomere elongation [119,197]. Furthermore, persistent R-loops trigger the DDR and promote Rad51-mediated homology-direct repair (HDR). In telomerase negative strains that also lack RNase H proteins—endonucleases that specifically degrades the RNA of RNA-DNA hybrids—R loops accumulated to promote HDR, and resulted in delayed senescence by about 25 population doublings [198]. However, this observed delay may not solely be due to the accumulation of TERRA at telomere ends, but rather could arise from effects of RNA-DNA hybrid accumulation throughout the genome as a result of RNase H inactivation. In human cells, less is known about the dynamics of TERRA and its contributions to senescence and aging. In telomerase-negative human cancer cells, which maintain telomeres using the homologous recombination-dependent ALT pathway, R-loops have been shown to play an essential role in telomere maintenance [199,200]. With persistent DNA damage or depletion of TRF2 leading to telomere deprotection, TERRA levels increase [201]. Apart from TRF2, TERRA also interacts with an array of proteins including other shelterin proteins, DNA replication proteins, cell cycle regulators and transcription factors. Of note, among these proteins, only TRF1 and TRF2 depletion affects TERRA levels [201]. On top of this, TERRA in humans is not restricted to the telomeres, but can localize throughout the genome, mainly at introns or chromosome regions within genes, where it contributes to gene expression regulation. Depletion of TERRA resulted in downregulation of subtelomeric genes, and general dysregulation at other target genes. The exact mechanisms of how TERRA regulates gene expression is unknown, though it could be possible that by binding near transcription start sites (a characteristic of genes downregulated upon TERRA depletion) and forming DNA-RNA hybrids, the nucleosome landscape is perturbed in favor of promoter clearance [202]. Interestingly, TERRA strongly colocalized with the helicase ATRX. However, TERRA and ATRX have functionally opposing roles at shared target genes, and whether ATRX roles in this context connect with its assistance to the histone chaperone DAXX have not yet been tested [202]. Altogether, these findings indicate that TERRA plays a major role in telomere maintenance and gene regulation, though more solid evidence is needed to link TERRA to aging and age-related pathologies. 

## 8. Conclusions 

For individuals fortunate enough to escape premature death by disease or accident, aging is an inevitable outcome of life, and continues to intrigue the scientific community. Epigenetic mechanisms are emerging as important and possibly tunable mediators of the aging process. Epigenome editing is an attractive avenue in therapeutics because, on the one hand, it has the advantages of being reversible compared to genetic manipulations, while, on the other hand, it is relatively stable and can be perpetuated through cell divisions. However, epigenetic regulators tend to have broad effects throughout the genome, and thus targeting particular genomic regions is challenging. Sequence-specific targeting of chromatin modifiers, e.g., by fusion to TALEN and Crispr-Cas9 proteins [203,204,205,206], may provide an approach to this problem, but delivery of such regulators to cells within intact tissues is currently a substantial barrier to most therapeutic uses. An overarching mechanism by which chromatin-based manipulations appear to promote longevity is via activation of stress responses. However, extending lifespan at the expense of exposure to increased stress may be undesirable. Therefore, it is in this regard that epigenetic manipulations might prove to be especially powerful, as it may be possible to edit the epigenome to mimic the salutary aspects of the “stressed” state without the negative effects of exposure to actual stresses.
